# Agreeableness modulates mental state decoding: Electrophysiological evidence

**DOI:** 10.1002/hbm.26593

**Published:** 2024-01-30

**Authors:** Elisabetta Pisanu, Sandra Arbula, Raffaella Ida Rumiati

**Affiliations:** ^1^ Neuroscience Area, SISSA Trieste Italy; ^2^ Dipartimento di Medicina dei Sistemi Università degli Studi di Roma “Tor Vergata” Rome Italy

**Keywords:** agreeableness, Big Five, electroencephalography, mental state decoding, personality, Reading the mind in the eyes, theory of mind

## Abstract

Agreeableness is one of the five personality traits which is associated with theory of mind (ToM) abilities. One of the critical processes involved in ToM is the decoding of emotional cues. In the present study, we investigated whether this process is modulated by agreeableness using electroencephalography (EEG) while taking into account task complexity and sex differences that are expected to moderate the relationship between emotional decoding and agreeableness. This approach allowed us to identify at which stage of the neural processing agreeableness kicks in, in order to distinguish the impact on early, perceptual processes from slower, inferential processing. Two tasks were employed and submitted to 62 participants during EEG recording: the reading the mind in the eyes (RME) task, requiring the decoding of complex mental states from eye expressions, and the biological (e)motion task, involving the perception of basic emotional actions through point‐light body stimuli. Event‐related potential (ERP) results showed a significant correlation between agreeableness and the contrast for emotional and non‐emotional trials in a late time window only during the RME task. Specifically, higher levels of agreeableness were associated with a deeper neural processing of emotional versus non‐emotional trials within the whole and male samples. In contrast, the modulation in females was negligible. The source analysis highlighted that this ERP‐agreeableness association engages the ventromedial prefrontal cortex. Our findings expand previous research on personality and social processing and confirm that sex modulates this relationship.

## Graphical abstract

Agreeableness personality trait affects brain's activity during mental state decoding, namely during the first stage of theory of mind. Subjects with high agreeableness show a deeper neural processing of emotional versus non‐emotional trials in a late time window and by mainly engaging the ventromedial prefrontal cortex.

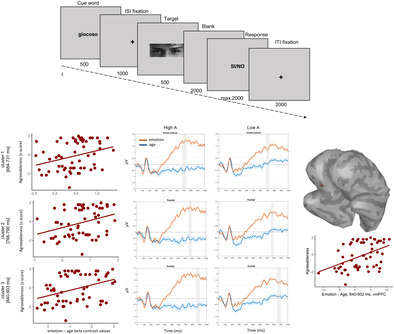

## 1 INTRODUCTION

The agreeableness personality trait of the five‐factor model (FFM) reflects individual differences in pro‐social tendencies, cooperation, empathy, and interpersonal harmony (Costa & McCrae, 1992; Goldberg, 1990). Agreeableness has been associated with neural processes involved in emotional regulation, social cognition, reward sensitivity, and theory of mind (ToM; DeYoung & Allen, 2017; DeYoung & Blain, 2020; Nettle & Liddle, 2008; Udochi et al., 2022). Individual variations in agreeableness seem to correspond to differences in social–emotional content processing. For instance, during a social animation task, individuals with lower levels of agreeableness exhibited more similar neural patterns of activation for social and non‐social content, whereas more agreeable individuals displayed more distinct neural patterns for these two information in the dorsomedial prefrontal cortex (Arbula et al., 2021). This latter brain region has often been reported in ToM (Sabbagh & Bowman, 2018; Saxe & Baron‐Cohen, 2006; Schurz et al., 2014, 2021).

Furthermore, to understand whether lower‐order processes, corresponding to early sensory and perceptual processing stages, or higher‐order processes encompassing slower inferential processes are affected, our previous electroencephalography (EEG) study targeted the detection of social interactions as one of the processes that could underlie agreeableness (Pisanu et al., n.d.). We argued that agreeableness affects social stimulus processing in a relatively early time window (~200 ms) but that this modulation depends on the sex of the participants. Specifically, in male participants agreeableness had an ample effect on social stimulus processing that, from the beginning of the trial, extends beyond the end of the stimulus presentation that lasted 500 ms, and was also predictive of their performance. Over and above the sex effect, individuals with lower levels of agreeableness displayed reduced responsivity to social stimuli compared with non‐social stimuli, while individuals with higher levels of agreeableness showed the opposite pattern.

What remains to be addressed is whether the modulation of agreeableness occurs for both social and emotional stimuli processing associated with ToM, and whether it is affected by the complexity of the task and of the neural processes analyzed.

To address these questions, in the present EEG study, the same participants as in Pisanu et al. (n.d.) performed two tasks of varying levels of complexity, specifically aimed at decoding emotional cues. The first task is the Reading the Mind in the Eyes (RME; Baron‐Cohen et al., 2001) that engages high processes of mental state recognition (i.e., compassionate, playful, confused) only through the expression of the eyes. It is a well‐established task of mental state decoding, representing the first stage of ToM that refers to the identification from visible cues of the type of mental state without encompassing the subsequent stage of inferring the content associated with that mental state, such as the compassion for a specific event like the loss of one's dear friend (Baron‐Cohen et al., 2001). As such it is thought to evaluate the subjects' ability to “put themselves into the mind” of the other person by attuning to their emotions. The second task we employed is the Biological (e)Motion (BM) task (Atkinson et al., 2012) that, unlike the RME task, requires lower emotional perception processes to discriminate basic emotional actions (i.e., happiness, anger, and sadness) expressed in the point‐light bodily movement stimuli. It is well established that bodily movements serve as a rich and immediate source of social information (Beall et al., 2008; De Gelder, 2006) that can be rapidly and effortlessly extracted even from sparse visual information, as effectively demonstrated in point‐light bodily stimuli (Blake & Shiffrar, 2007; Puce et al., 2015). Extracting social information from bodily movements of others has been proposed to rely on the sensorimotor system, suggesting the possible involvement of a simulation process. This system is the same that is involved in action execution (Del Vecchio et al., 2020; Gallese & Goldman, 1998; Jeannerod, 2001, 2006; Rizzolatti & Craighero, 2004). For instance, EEG recordings consistently reveal a decrease in alpha‐band (8–12 Hz) oscillations over the sensorimotor cortex. This phenomenon, known as μ‐alpha suppression, is argued to reflect the mental simulation of action and has been proposed to facilitate the internal representation of others' emotional states (see Fox et al., 2016; Hobson & Bishop, 2017 for a review). Using point‐light body stimuli, Siqi‐Liu et al. (2018) observed a more pronounced μ‐suppression in response to emotional actions compared to neutral actions, indicating a heightened neural response to actions involving an emotional component from the middle to the end of the stimulus presentation. Furthermore, these authors found that individuals characterized by lower levels of autistic trait demonstrated a more pronounced degree of μ‐suppression in comparison to those with higher levels of the same trait.

In this study, using these two tasks enabled us to test the interplay between early‐stage emotional perception and higher‐level mental state inference. In doing so, we could then provide a comprehensive understanding of the relationship between personality and the neural mechanisms underlying emotional cue decoding, spanning from the perception of basic emotional actions to the recognition of complex mental states. We hypothesized that variations in the levels of agreeableness would correlate with differences in the ability to detect emotions, along with modulations in event‐related potential (ERP) responses in the emotional condition compared with the non‐emotional condition. We also hypothesized a modulation of event‐related spectral perturbations (ERSP) responses expressed as a stronger μ‐suppression for more agreeable participants.

We also predicted that this modulation would depend on the type of task, with a more extensive and later influence of agreeableness for the RME task than the BM task. Particularly, since the former task seems to involve more complex neural processes necessary to recognize complex mental states, while the latter employs stimuli like biological motion and basic emotions that typically require automatic processing without conscious awareness and attentional cognitive resources (e.g., Celeghin et al., 2017; Del Piero et al., 2016; Nakashima et al., 2023; Simion et al., 2008; Winkielman & Hofree, 2012), we hypothesized that the modulation of agreeableness on the neural processes underlying these two tasks might emerge as temporally distinct. Furthermore, as in our previous EEG study where we found a sex effect in agreeableness (Pisanu et al., n.d.), we expected that the emotional processes at study here should be modulated by sex.

Finally, we measured the impact of agreeableness on the spontaneous processing of gaze stimuli. We reasoned that individuals with higher agreeableness may exhibit heightened sensitivity to eye stimuli even during passive observation, without engaging in any specific actions or decision‐making processes. Considering that agreeableness seems to foster a heightened attentional and motivational orientation toward social stimuli (Moore et al., 2014; Pisanu et al., n.d.; Wilkowski et al., 2006), and that eyes are essential social stimuli conveying crucial emotional messages, it is plausible that individuals with higher agreeableness may be more sensitive to such stimuli per se. Thus, we predicted that variations in the levels of agreeableness would correlate negatively with differences in processing gaze stimuli during passive and active observation, reflecting more similar neural responses between the two conditions in individuals high in agreeableness, and more distinct neural responses in individuals low in agreeableness.

This study extends our previous research on how agreeableness modulates social–emotional processes associated with ToM. Notably, among the Big Five personality traits, only agreeableness is distinctly linked to ToM (e.g., Allen & DeYoung, 2017; DeYoung & Blain, 2020). Consequently, in line with our focused hypothesis, participants were intentionally pre‐selected based on their agreeableness scores.

## 2 MATERIALS AND METHODS

### 2.1 Participants recruitment and personality assessment

Here, 62 young adults (27 males, mean age 24.03, SD = 4.34, range = 18–35 years) were selected from a larger sample (*N* = 462) previously screened for agreeableness to increase in‐sample variance by means of the Italian adaptation of the Big Five Inventory (Ubbiali et al., 2013) administered online: 22 participants had scores below, and 20 above 1.4 standard deviations from the Italian population mean, while the remaining 20 had scores around the average. As personality traits are continuous variables, in order to ensure that our sample accurately captures measurements at either tail of the distribution, deliberate efforts were made to select individuals with diverse scores along the z‐score continuum, encompassing both negative and positive values. This sampling strategy increases the power to detect moderate effect sizes in small samples, without inflating the false positive rate (de Haas, 2018). The questionnaire consists of 44 items rated on a 5‐point Likert scale aimed at evaluating the five personality traits from the FFM (extraversion, neuroticism, openness, conscientiousness, and agreeableness). Participants were recruited through social media and email. Exclusion criteria included a history of neurological or psychiatric disorders. All participants provided written informed consent and received 20 euros upon completion of the study. The study and informed consent procedures were approved by the SISSA's ethics committee and adhered to the guidelines of the Declaration of Helsinki.

### 2.2 Reading the mind in the eyes task

This task is an adaptation of The Reading the Mind in the Eyes (Baron‐Cohen et al., 2001) for use in an ERP paradigm. Participants were presented with an Italian cue word followed by a target image. The cue could either represent one of the complex emotional states from the original paper and pencil task (e.g., playful), an age band (e.g., 29–39 years) or the word “observe.” Targets were represented by 36 black and white photos of the eyes of male or female individuals from the original task. In both the emotion and age conditions, after each target, participants were asked to indicate whether according to them the cue was congruent with the target by pressing a key button (yes/no). In the “observe” condition, participants were instructed to simply watch the image without taking action.

Previous neuroimaging studies adopted the sex decoding as the control task (Sabbagh et al., 2004) nevertheless, this option entails a significant constraint, namely that the two conditions exhibit a pronounced imbalance, with the control condition (sex decoding) being excessively easy in comparison to the experimental condition (emotional state decoding) affecting the interpretation of the results especially in terms of ERP components involved (Sabbagh et al., 2004). Therefore, we chose a more difficult condition, namely the age decoding, first piloted behaviorally in a sample of 31 subjects (see Supplementary materials for the behavioral pilot results of the RME task). Furthermore, we also added the “observe” condition to analyze possible individual differences emerging even simply by watching gaze stimuli without acting.

**FIGURE 1:**
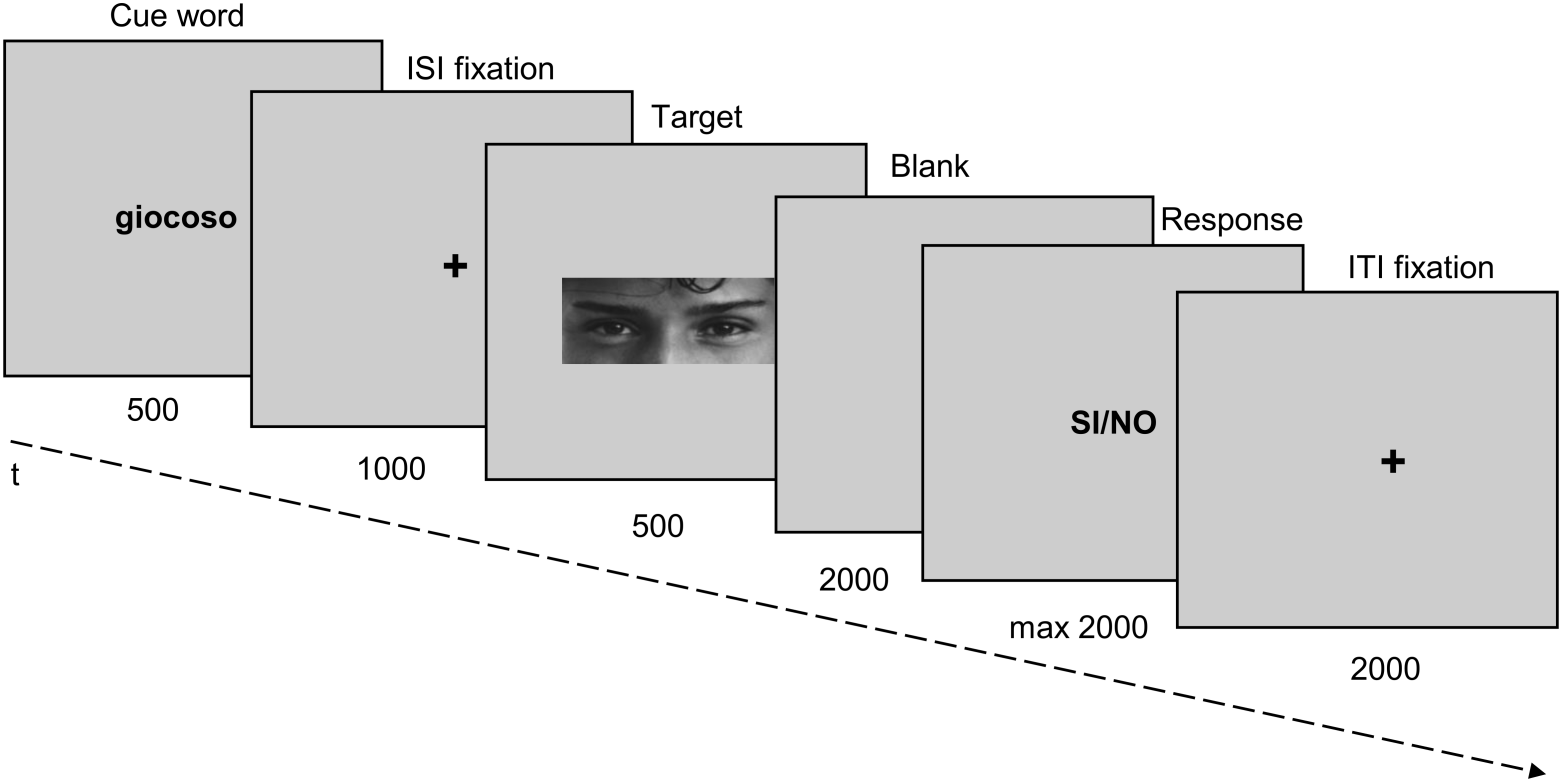
Schematic representation of the “reading the mind in the eyes task”. Stimuli succession is represented left to right, with the respective screen presentation time expressed in milliseconds below each stimulus.

Participants were seated 60 cm from the screen in a dark and quiet room. The stimuli were presented at 9 × 5 degrees of visual angle on a grey background, in the center of the screen by using PsychoPy3 (v2020.1.1). The task started with four practice trials containing emotional states and age bands (these responses were not included in the analyses). Participants completed 150 trials (50 per condition: emotion, age, observe) interspersed with a self‐paced pause every 50 trials. Stimuli were arranged in a pseudorandom order to avoid more than two repetitions of the same condition. Each trial began with the presentation of the cue (500 ms), followed by a fixation cross (1000 ms), and the target image (500 ms); after the offset of the target, participants were presented with a blank screen (2000 ms) after which they had 2000 ms to give their evaluation via button press (Figure 1). To respond, participants pressed either the “f” or the “j” key on a QUERTY keyboard with the index finger of both hands; additionally, response keys were counterbalanced between subjects. The evaluation was absent during the “observe” condition.

### 2.3 Biological (e)motion task

Participants were presented with an Italian cue word followed by a target video. The cue could either represent one of the five basic emotions (happiness, sadness, fear, disgust, anger) or a common everyday action (knocking, hopping, digging, walking on spot, star jumping, touching toes). Targets were represented by 11 whole‐body actions depicted in point‐light displays videos, 5 representing an emotional action, and 6 representing emotionally neutral actions. After each target video, participants were asked to indicate whether according to them the cue was congruent with the target by pressing a key button (yes/no). The stimuli were provided by Atkinson et al. (2012).

**FIGURE 2:**
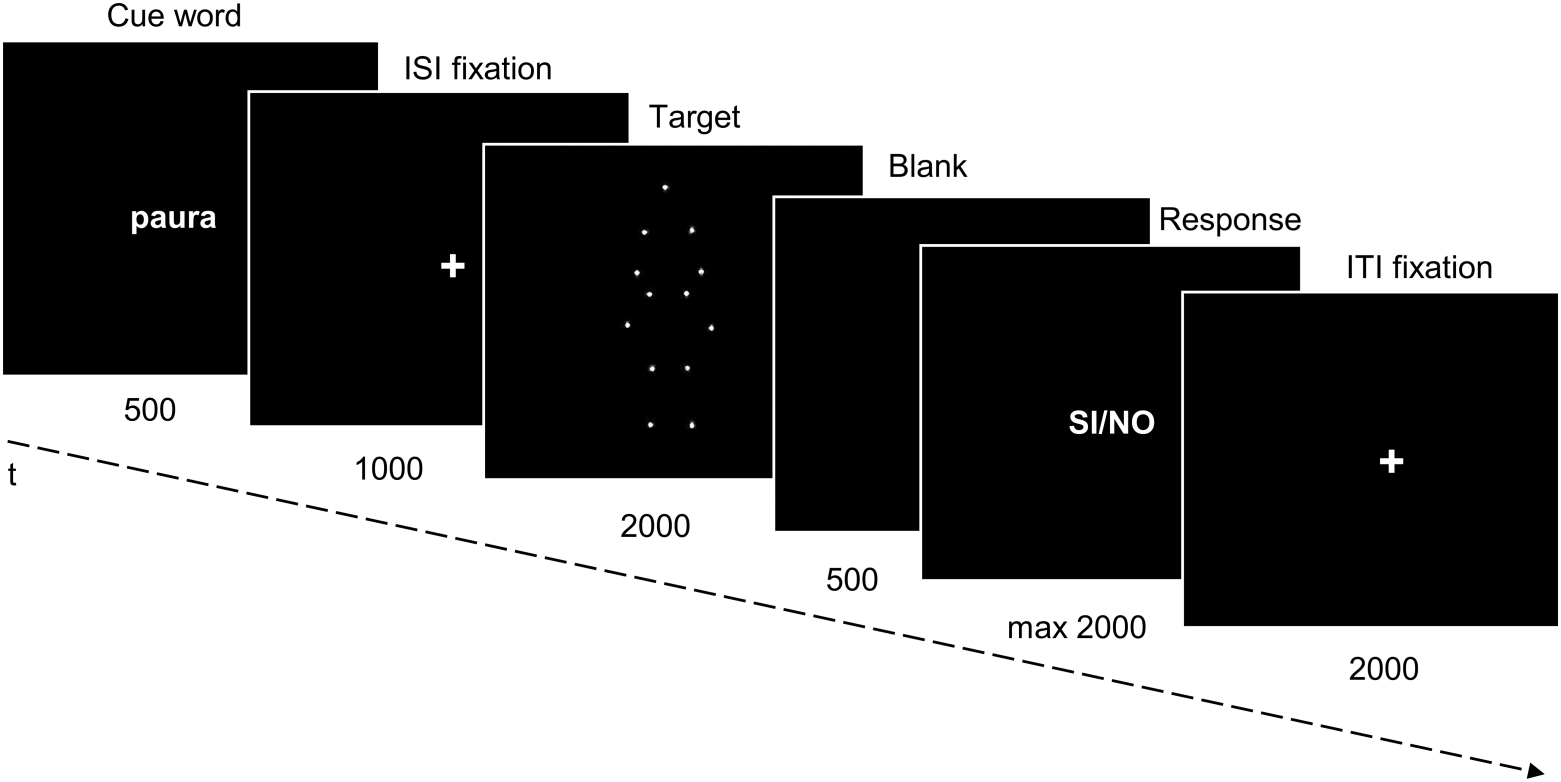
Schematic representation of the “biological (e)motion task”.

Participants were seated 60 cm from the screen in a dark and quiet room. The stimuli were presented at 9 × 5 degrees of visual angle on a black background, in the center of the screen by using PsychoPy3 (v2020.1.1). The task started with four practice trials containing emotional and neutral actions (these responses were not included in the analyses). Participants completed 100 trials (50 per condition) interspersed with a self‐paced pause after 50 trials. Stimuli were arranged in a pseudorandom order to avoid more than two repetitions of the same condition. Each trial began with the presentation of the cue (500 ms), followed by a fixation cross (1000 ms), and the target video (2000 ms); after the offset of the target, participants were presented with a blank screen (500 ms) after which they had 2000 ms to give their evaluation via button press (Figure 2). The response procedure is identical to that employed in the RME task.

### 2.4 EEG recording and preprocessing

EEG data were recorded using a 64 Ag/AgCl active electrode setup based on the International 10–20 system (Klem et al., 1999), and signal amplification was achieved with a BioSemi Active‐Two amplifier system. The ActiView acquisition software (ActiView 707, Biosemi, Amsterdam, The Netherlands) was adopted for visualization and data storage. Electrode offsets were maintained within ±50 mV, and the signal was sampled at 1024 Hz with 24‐bit digitization resolution. Eye movements and blinks were monitored using horizontal and vertical electrooculogram recorded from additional electrodes placed below and above the left eye.

Offline data preprocessing was performed using EEGLAB 14.1.1b (Delorme & Makeig, 2004, https://sccn.ucsd.edu/eeglab) in Matlab R2021a (https://uk.mathworks.com/products/matlab.html). The continuous EEG recordings were resampled at 250 Hz and band‐pass filtered (0.25–45 Hz) using zero‐phase Hamming windowed sinc FIR filters (transition bandwidth: 0.5–10 Hz, passband edges: 0.5–40 Hz). Poorly correlated channels were identified and removed using the EEGLAB *clean_rawdata* plugin (autocorrelation parameter = 0.8, resulting in an average exclusion of 1.9 channels, SD = 2.1). Epochs ranging from 1000 ms before to 2500 ms after target onset were extracted. Independent component analysis (Makeig et al., 1996) was employed to remove components with more than 60% probability of being eye, motor, channel noise, heart, or line noise using ICLabel (Pion‐Tonachini et al., 2019).

Removed channels were replaced using spherical spline interpolation (Perrin et al., 1989), and data were re‐referenced to a common average reference. Epochs were baseline‐corrected to a 200 ms pre‐stimulus baseline and inspected for artifacts based on different statistics (amplitude, linear trend, joint probability, and kurtosis) (Delorme et al., 2007). Overall, 10% of the RME trials and 8% of the BM ones were rejected for violating artifact criteria (with no asymmetry across conditions). The average number of epochs used for the analysis was: 45 in the emotion and age conditions, and 44 in the observe condition for the RME, ranging from 33 to 49 epochs; 46 in both conditions for the BM, ranging from 37 to 50 epochs. For the former task, three participants (one male and two females) were excluded from the EEG analysis due to technical problems during the recording.

Afterward, the ERSP were extracted from the artifact‐free ERP epochs (−1000–2500 ms) by means of a Morlet wavelet convolution (Herrmann et al., 2014). We estimated 42 log‐spaced frequencies, ranging between 4 and 45 Hz, using 4 cycles at lowest frequency to 22.5 at highest, with a temporal resolution of approximately 12 ms. The baseline correction was performed relative to the average power in the time interval between 900 and 400 ms prior to stimulus onset.

### 2.5 Behavioral data analysis

We collected accuracy and reaction times (RTs) data. RTs were filtered for errors, anticipated responses (<150 ms) and outliers above 3 standard deviations from the subjects mean for each condition. We used R v.4.1.1 (R Core Team, 2020) to perform linear mixed models selecting accuracy and RTs separately as dependent variables, type of condition (i.e., emotional, non‐emotional) and agreeableness as two fixed effects, and subject intercepts as random effects. To explore potential sex differences in the relationship between agreeableness and performance, two other models were developed (one for accuracy and one for RTs) by including sex as an additional fixed effect. We also checked for sex differences in agreeableness scores by means of an independent *t* test. To ensure that any observed effects were specific to agreeableness and not influenced by other personality traits, the other four traits were then included as additional predictors in the models. Assumptions for the models were checked through the visual inspection of residual plots (linearity, homoscedasticity, and normality).

### 2.6 ERP analysis

ERP analyses were carried out separately for the RME and BM task by using Matlab R2021a along with EEGLAB 2020_0 and LIMO EEG plugin (v 3.3, https://github.com/LIMO-EEG-Toolbox/limo_tools) that allows for performing robust statistics, handling non‐normal data and outliers. A massive univariate approach was adopted by performing two levels of analysis for both the two tasks (Pernet et al., 2011).

#### 2.6.1 First‐level GLM analysis

At the first level, parameters of a general linear model were estimated by using a standard ordinary least squares solution for each subject, at each time point and each electrode independently, and separately for all the experimental conditions. To assess the relationship between agreeableness and the differential activity elicited by the experimental conditions, the corresponding ERP amplitudes were subtracted to create a contrast (emotion—age, and emotion—observe for the RME task; emotional action—neutral action for the BM task). The selected time windows ranged from 0 to 1000 ms for the RME task, and from 1000 to 2500 ms for the BM task, time locked to stimulus‐target onset. For the latter task, in which the stimulus had a longer duration being a video, in line with the findings of Siqi‐Liu et al. (2018), we started the analysis from 1000 ms discarding the time points related to early neural processes which were not our target, and instead keeping the time points following the end of the stimulus as for the RME task. Erroneous trials were retained in the model to preserve higher interindividual variability, as we predicted that accuracy would vary based on agreeableness scores.

#### 2.6.2 Second level GLM analysis

At the second level, for both tasks, robust paired *t* tests between emotional and non‐emotional conditions were carried out across all subjects, all time points, and all electrodes in order to compare participants' ERP amplitudes across different conditions. Results are reported corrected for multiple testing using the maximum statistics thresholding *p* = .01 (Maris & Oostenveld, 2007; Pernet et al., 2015) that ensures robust control over the family‐wise error rate, providing both reliable significance and precise spatio‐temporal localization of the effect observed (Groppe et al., 2011a, 2011b). Afterward, robust skipped Pearson correlations were performed (Wilcox et al., 2018) by means of the robust correlation toolbox (https://github.com/CPernet/robustcorrtool; Pernet et al., 2013) between the agreeableness scores and the ERP contrast values (emotional—non‐emotional) of each subject in the time points and electrodes that resulted significant from the abovementioned paired *t* tests. These robust correlations were designed to handle data that deviate from normality and/or outliers; particularly, unlike parametric correlations, here the influence of bivariate outliers is downweighted, making it more robust in the presence of extreme data points. The statistical significance was tested through the percentile bootstrap 95% confidence intervals and results are reported with the FDR correction for multiple comparisons. The same correlation analyses were repeated separately for the samples of males and females to unravel potential sex differences. Significant correlation effects that lasted <20 ms were not reported.

By running the robust correlation based on the results from the paired *t* test, we were able to bypass the preselection of specific time intervals and electrodes, while still performing a targeted investigation which considers the most relevant and informative data for the relationship between agreeableness and neural responses. This approach minimized the likelihood of false positive findings and enhanced the interpretability of the results. Additionally, the use of robust correlations reduced the impact of outliers by considering the overall structure of the data and excluding bivariate outliers.

### 2.7 ERSP analysis

Since our hypothesis specifically targeted a possible association between agreeableness and μ‐alpha suppression, ERSP analysis was performed only for the BM task by using Matlab R2021a along with EEGLAB 2021_0 and LIMO EEG plugin (v 3.3). As for the ERPs, a massive univariate approach was used by running two levels of analysis (Pernet et al., 2011).

#### 2.7.1 First‐level GLM analysis

At the first level, parameters of a General Linear Model were estimated for each subject, at each time point, each electrode and each frequency in the range between 8 and 20 Hz independently, and separately for the experimental conditions, namely emotional action and neutral action. The time window ranged from 0 to 2500 ms time locked to stimulus onset, excluding edge artifacts induced by the wavelet transform. Parameters were estimated by using a standard ordinary least squares solution.

#### 2.7.2 Second level GLM analysis

At the second level, a robust paired *t* test between the emotional and emotionally neutral actions conditions was carried out across all subjects, all time points, all electrodes, and all frequencies. Results are reported corrected for multiple testing using spatial–temporal clustering *p* = .01 (Maris & Oostenveld, 2007; Pernet et al., 2015).

Afterward, we performed a robust skipped Pearson correlation between the ERSP contrast values (emotional action—emotional‐neutrally action) of each subject and their agreeableness scores in the time points, electrodes and averaged range of frequencies that resulted significant in the abovementioned paired *t* test. Frequencies were averaged to reduce the dimensionality of the data and consequently the number of multiple tests. Correlations were performed within the entire, female and male sample. Results are reported corrected for multiple testing using the FDR.

Hence, we applied the same methodology as employed in the ERP analyses for the reasons outlined above and to ensure methodological consistency for clearer result interpretation. This approach is especially useful for examining the μ‐suppression effect. Indeed, while the common practice of a priori selection of central electrodes is prevalent, it introduces the risk of circularity in analyses, hindering efforts to shed light on the complexities of this phenomenon (Hobson & Bishop, 2016; Hobson & Bishop, 2017).

### 2.8 Source analysis

The estimation of cortical source activations was performed using the Brainstorm software, which adopts a distributed dipoles model for fitting (Tadel et al., 2011). To determine the locations of the recorded electrical activity on the scalp, the dynamic statistical parametric mapping method was employed, which incorporates minimum‐norm inverse maps (Dale et al., 2000). For each participant, single subject noise covariance matrices and individual noise standard deviations at each location were calculated and estimated based on the pre‐stimulus baseline intervals (−200 to 0 ms) from single trials (Hansen et al., 2010).

To build the head model, the boundary element method (BEM) was utilized with default parameters in Brainstorm, using the ICBM152 anatomy, as individual anatomies were unavailable. The BEM model provides three realistic layers and representative anatomical information (Gramfort et al., 2010; Stenroos et al., 2014). The source estimation was carried out adopting the option of constrained dipole orientations, where one dipole‐oriented perpendicular to the cortical surface was modeled for each vertex (Tadel et al., 2011).

To estimate active sources, single‐trial EEG data for each participant were averaged to obtain the subject average. Sources were then estimated separately for the experimental conditions (emotional and non‐emotional) and subtracted to create a contrast at the subject level for both tasks. Z‐score normalization was applied to each source, utilizing the baseline period (−102 to −4 ms), to mitigate the influence of interindividual fluctuations in neural current intensity resulting from nonrelevant anatomical or physiological differences, and rectified them in absolute values.

Afterward, we computed paired *t* tests between emotional and non‐emotional conditions across subjects in the time windows‐averaged of the ERP effects. Accordingly, for the RME task, two paired *t* test were performed: emotion—age and emotion—observe. We then selected the brain regions emerging from these statistics and used them as regions of interest (ROIs) to perform robust skipped Pearson correlations with agreeableness scores within the entire, female and male sample. The ROIs were defined manually by visual inspection to be as accurate as possible since no atlas provided could encompass them properly. Since we were interested in investigating the cortical sources underlying the ERP or ERSP effects associated with agreeableness within both tasks, statistics on sources were computed only when these effects were found.

## 3 RESULTS

### 3.1 Behavioral results

#### 3.1.1 Reading the mind in the eyes

**FIGURE 3:**
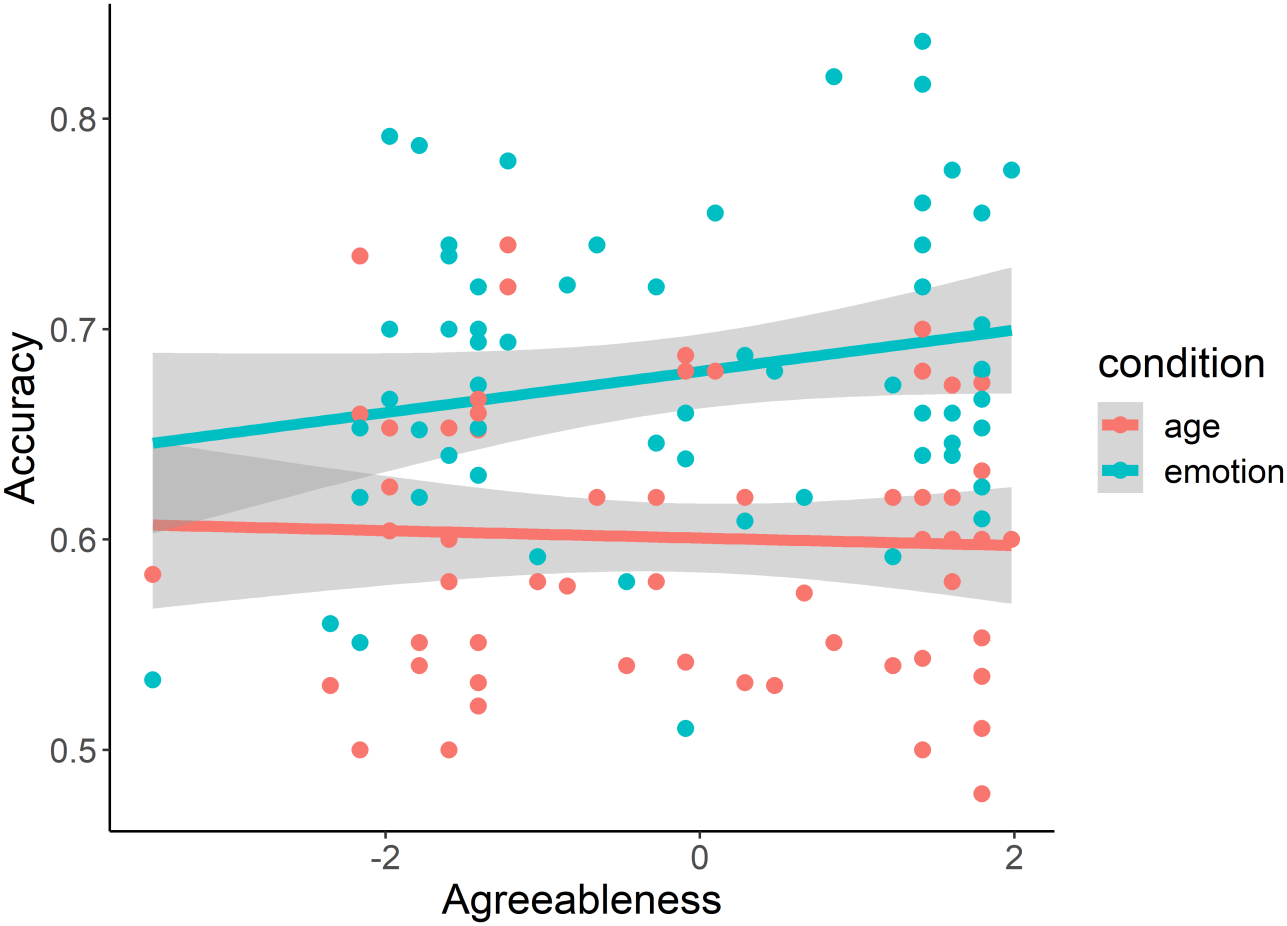
The scatterplot shows the relationship between agreeableness (represented in z scores on the x axis), and accuracy (on the y axis) in the reading the mind in the eyes (RME) task; the blue line represents emotion condition, the red line age's one; each spot is a subject. The grey shadow around the trendline represents the standard error (statistics and *p* values are reported in the text).

The linear mixed model testing whether agreeableness affects participants' performance in recognizing complex emotional states compared to age bands, captured by the accuracy measure, showed a trend suggesting that higher agreeableness scores predicted better ability to recognize emotions versus age, although the *p* value did not reach the significance (*F*(1, 60) = 3.23, *p* = .077, *η* ^2^ = .05; Figure 3). When including sex as a second fixed effect in the model, the interaction with agreeableness remained nonsignificant (*F*(1, 58) = 1.61, *p* = .21, *η* ^2^ = .03). No significant results were observed in the linear mixed models considering RT measure as a dependent variable, either with or without the consideration of sex (respectively: *F*(1, 60) = 2.06, *p* = .16, *η* ^2^ = .04; *F*(1, 58) = 3.04, *p* = .09, *η* ^2^ = .04). Males and females did not differ significantly in terms of agreeableness score (*t* = 0.08, df = 58.38, *p* = .94). No significant or nearly significant associations with any of the dependent behavioral variables investigated were observed for any of the other personality traits (accuracy: all *p*'s > .2; RTs: all *p*'s > .7).

#### 3.1.2 Biological (e)motion

No significant results emerged from the linear mixed models carried out on accuracy (agreeableness model: *F*(1, 60) = .69, *p* = .41, *η* ^2^ = 01; agreeableness × sex model: *F*(1, 58) = .77, *p* = .39, *η* ^2^ = .01) and RTs (agreeableness model: *F*(1, 60) = .98, *p* = .33, *η* ^2^ = .02; agreeableness × sex model: *F*(1, 58) = .03, *p* = .87, *η* ^2^ = .0004). No significant or nearly significant associations with any of the dependent behavioral variables investigated were observed for any of the other personality traits (accuracy: all *p*'s > .3; RTs: all *p*'s > .2).

Visual inspection of residual plots did not reveal any obvious deviations from linearity, homoscedasticity, or normality in both tasks.

### 3.2 ERP results

#### 3.2.1 Reading the mind in the eyes

**FIGURE 4:**
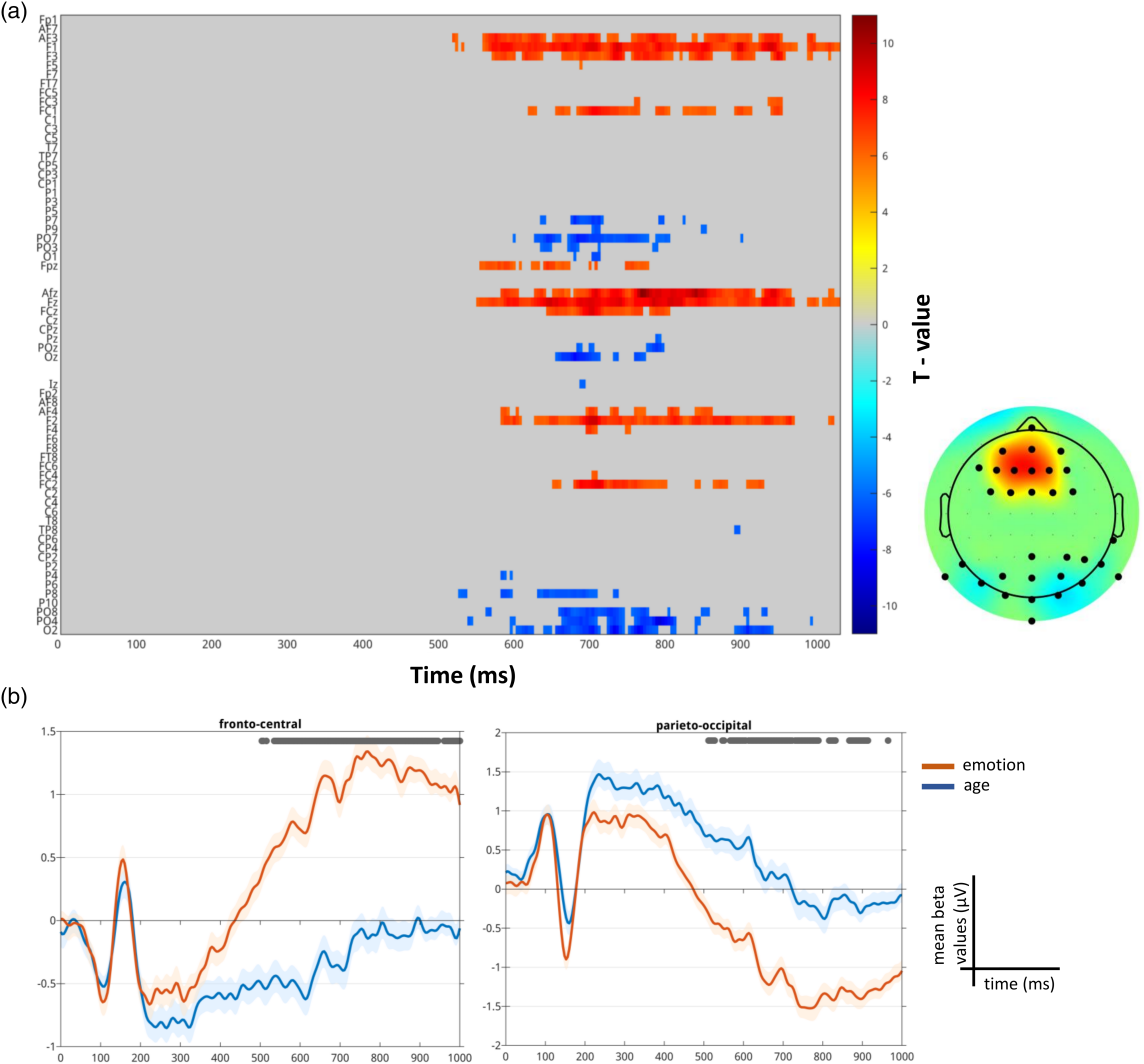
Event‐related potential (ERP) results from the paired *t* test (emotion—age), reading the mind in the eyes (RME) task. (a) Raster plot depicting significant channels and time‐points for the paired *t* test colored based on their *t* values. The electrodes on the y‐axis are arranged topographically: left and right sides of the head are organized, respectively, on the top and the bottom part of the axis, midline electrodes are displayed in the middle. Thus, overall the y‐axis represents the scalp anterior to posterior. On the right the topographical representation of the *t* values averaged across the significant time‐window (504–1000 ms); the larger black circles on the topographic plot represent the electrodes that formed the significant clusters. (b) Trace plots showing the mean beta values for the emotion (in red) and age conditions (in blue) averaged over the significant electrodes. The grey line at the top indicates the significant time window.

A significant difference emerged between the ERP neural response associated with the emotion condition and the age condition from the paired *t* test starting at 504 ms and ending at 1000 ms (max *t* value 10.73 at 746 ms on channel AFz, corrected *p* value .001; Figure 4a) within the entire sample. This difference was led by the emotional condition eliciting a higher neural response than the age condition in the abovementioned time window expressed by a larger late positivity over fronto‐central electrodes and, as a consequence of the dipole effect, a larger negativity over parieto‐occipital electrodes (Figure 4b).

**FIGURE 5:**
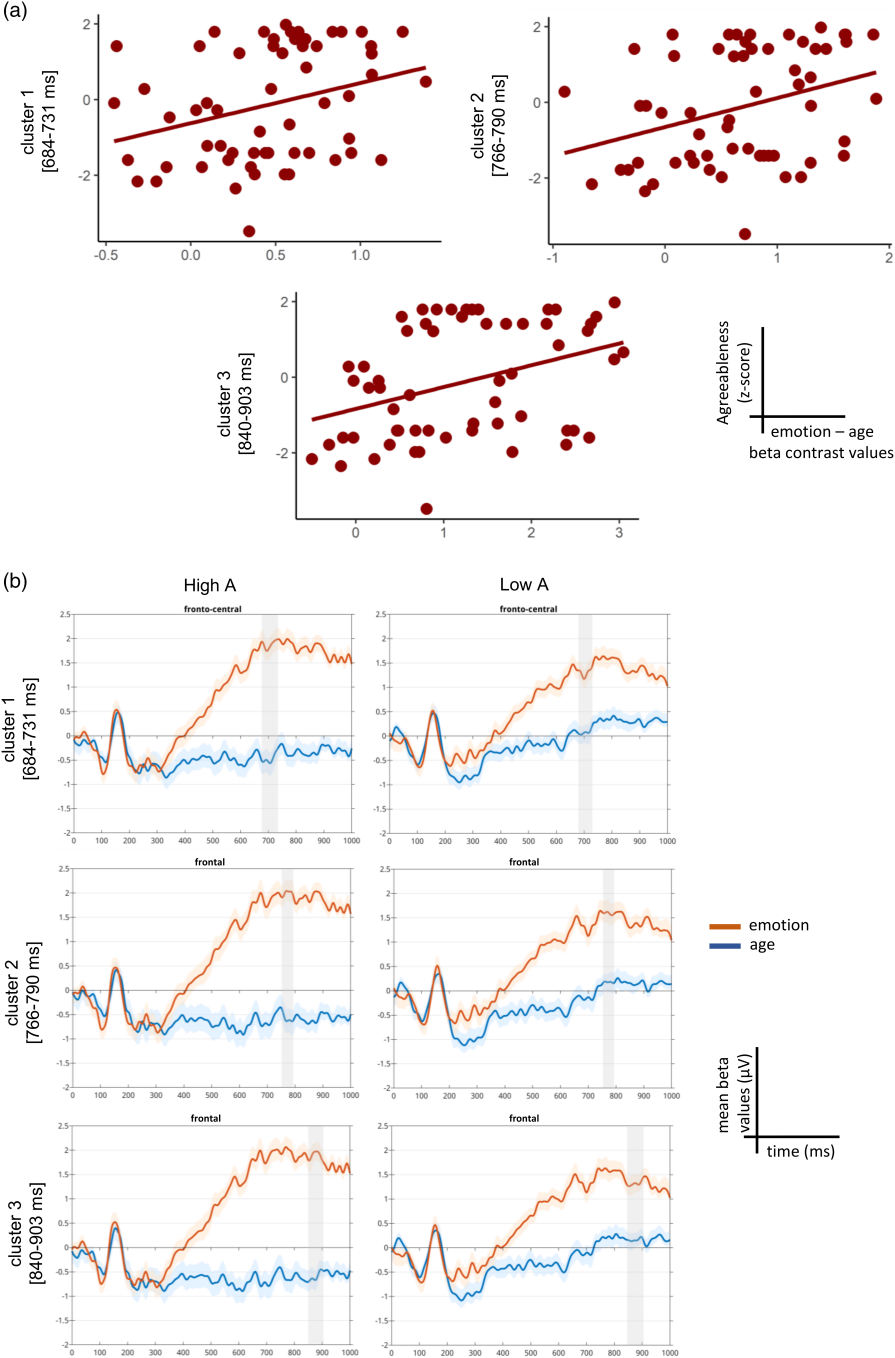
Event‐related potential (ERP)‐agreeableness skipped Pearson correlation results for the entire sample. (a) Scatterplots representing the correlation between the beta contrast values averaged across the significant time window and the agreeableness trait, separately for the three temporal clusters. (b) Trace plots showing the mean beta values for the emotion (in red) and age conditions (in blue) averaged over the significant electrodes from each of the three clusters, separately for the high A and low A group.

After performing the skipped Pearson correlation between agreeableness and the ERP emotion—age contrast values within the entire sample, we found three significant temporal clusters. Cluster 1 starts at 684 ms and ends at 731 ms on channels F1, Fz, F2, FC2, FCz (median *r* = .35, range = .28 to .46; median lower bound of the CI_95%_ = .14, range = .11 to .21; median upper bound of the CI_95%_ = .54, range = .51 to .60; all corrected *p* value < .05); cluster 2 starts at 766 and ends at 790 ms on channels F1 and Fz (median *r* = .47, range = .42 to .48; median lower bound of the CI_95%_ = .12, range = .09 to .14; median upper bound of the CI_95%_ = .56, range = .54 to .58; all corrected *p* value < .05); cluster 3 starts at 840 ms and ends at 903 ms on channels F1, Fz, F2 (median *r* = .40, range = .24 to .54; median lower bound of the CI_95%_ = .16, range = .12 to .23; median upper bound of the CI_95%_ = .58, range = .53 to .63; all corrected *p* value < .05). These results showed that agreeableness modulates the differential neural response such that subjects with higher agreeableness scores had more positive contrast values over the fronto‐central electrodes (Figure 5a). To inspect the direction of these contrasts across different levels of agreeableness, we divided the entire sample in two groups (high A: high agreeableness, low A: low agreeableness) by means of the median‐split of their agreeableness z‐scores, and plotted their averaged ERPs (i.e., first level beta values) for the emotion and age condition (Figure 5b). The plot reveals that the high A group exhibited a larger positive amplitude for emotion versus age trials over the fronto‐central electrodes and a larger difference between the two conditions with respect to the low A group. This pattern was the same for all the three temporal clusters, although the modulation of agreeableness was more extensive within the third and latest cluster where we observed the highest differentiation between the two conditions in the high A group with respect to the low A group.

As regards the female sample, no significant correlation effects that lasted > = 20 ms were observed.

**FIGURE 6:**
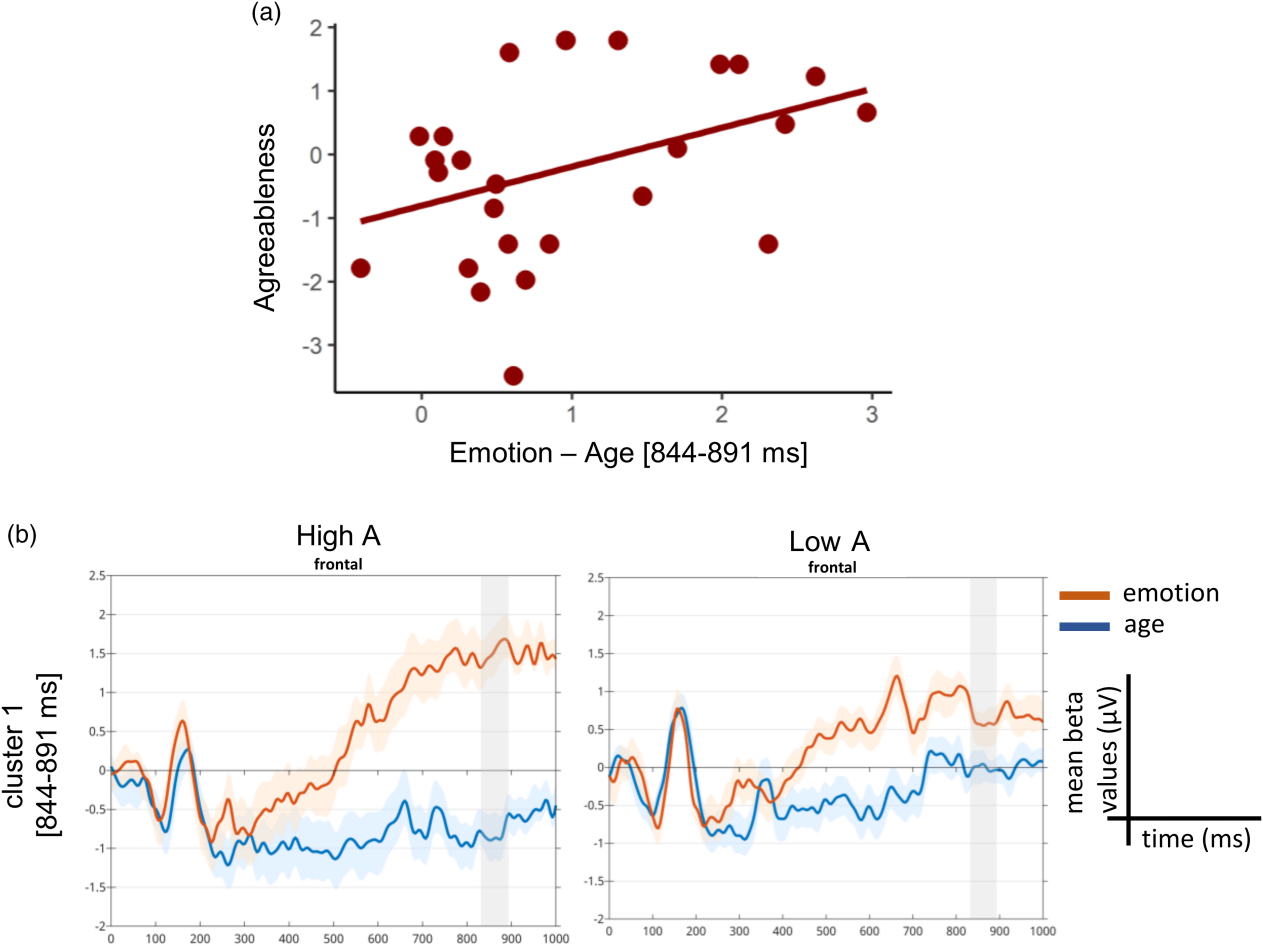
Event‐related potential (ERP)‐agreeableness skipped Pearson correlation results for the male sample. (a) Scatterplot representing the correlation between the beta contrast values averaged across the significant time window and the agreeableness trait for the significant temporal cluster. (b) Trace plots showing the mean beta values for the emotion (in red) and age conditions (in blue) averaged over the significant electrodes, separately for the high A and low A group.

Within the male sample, the robust correlation unveiled one significant temporal cluster starting at 844 ms and ending at 891 ms over channels AF3, F1, Fz (median *r* = .51, range = .43 to .57; median lower bound of the CI_95%_ = .18, range = .14 to .26; median upper bound of the CI_95%_ = .65, range = .63 to .76; all corrected *p* value < .05). Noteworthy, this cluster matched the third cluster found within the entire sample. Particularly, males with higher levels of agreeableness showed more positive contrast values over frontal electrodes than males with low agreeableness (Figure 6a). This result was driven by a greater difference between the ERPs associated with the two conditions, particularly guided by a larger positive amplitude for emotion versus age trials in more agreeable males compared to less agreeable ones (Figure 6b).

**FIGURE 7:**
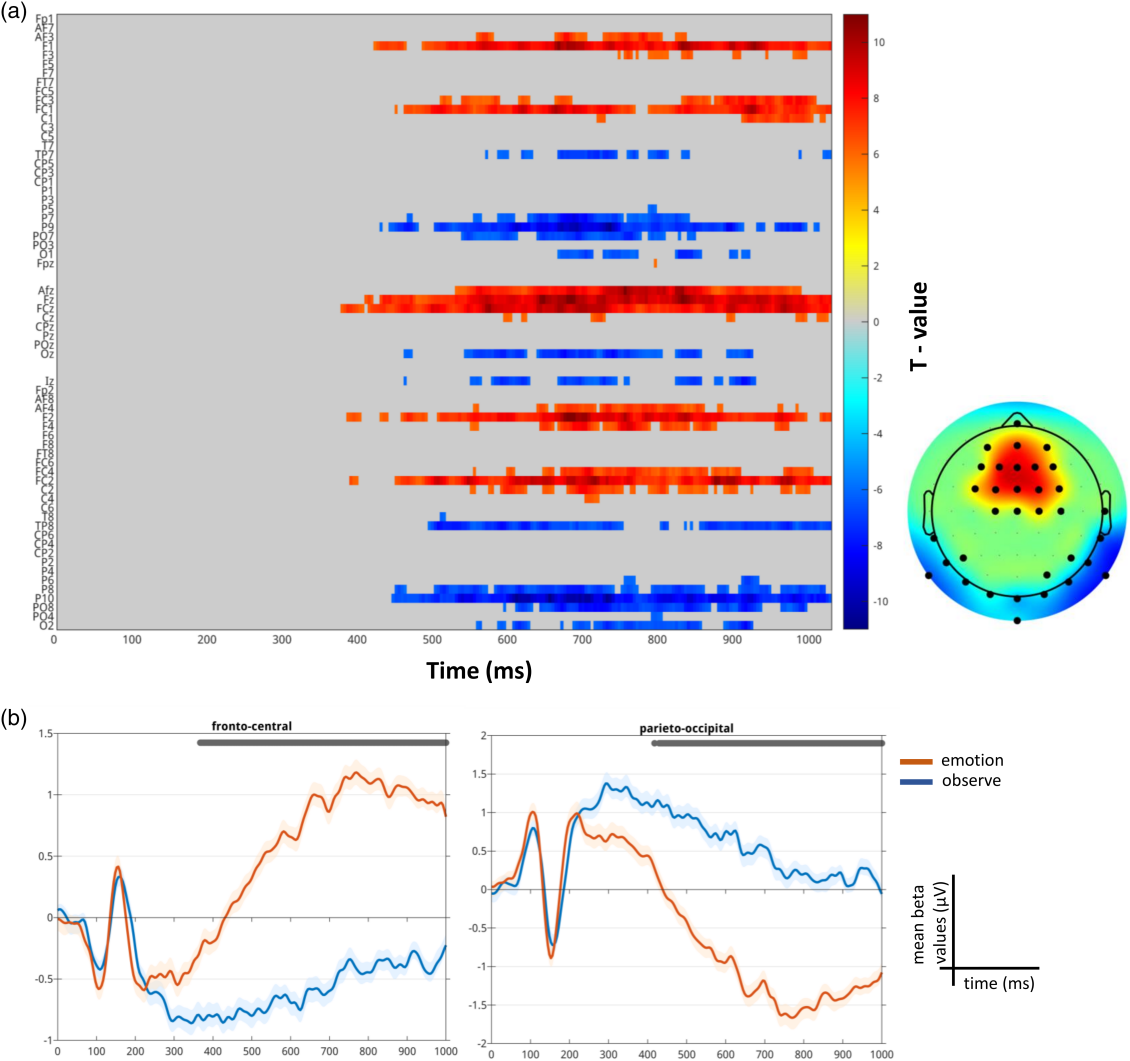
Event‐related potential (ERP) results from the paired *t* test (emotion—observe), reading the mind in the eyes (RME) task. (a) Raster plot depicting significant channels and time‐points for the paired *t* test colored based on their *t* values. On the right, the topographical representation of the *t* values averaged across the significant time‐window (367–1000 ms). (b) Trace plots showing the mean beta values for the emotion (in red) and observe conditions (in blue) averaged over the significant electrodes.

From the paired *t* test comparing the emotion and observe conditions, a significant neural difference emerged within the entire sample starting at 367 ms and ending at 1000 ms (max *t* value 11.37 at 664 ms on channel Fz, corrected *p* value .001; Figure 7a). This difference was explained by the emotional condition eliciting a higher neural response (larger late positivity) than the observe condition in the abovementioned time window over fronto‐central electrodes, and, as a consequence of the dipole effect, a larger negativity over the parieto‐occipital electrodes (Figure 7b).

**FIGURE 8:**
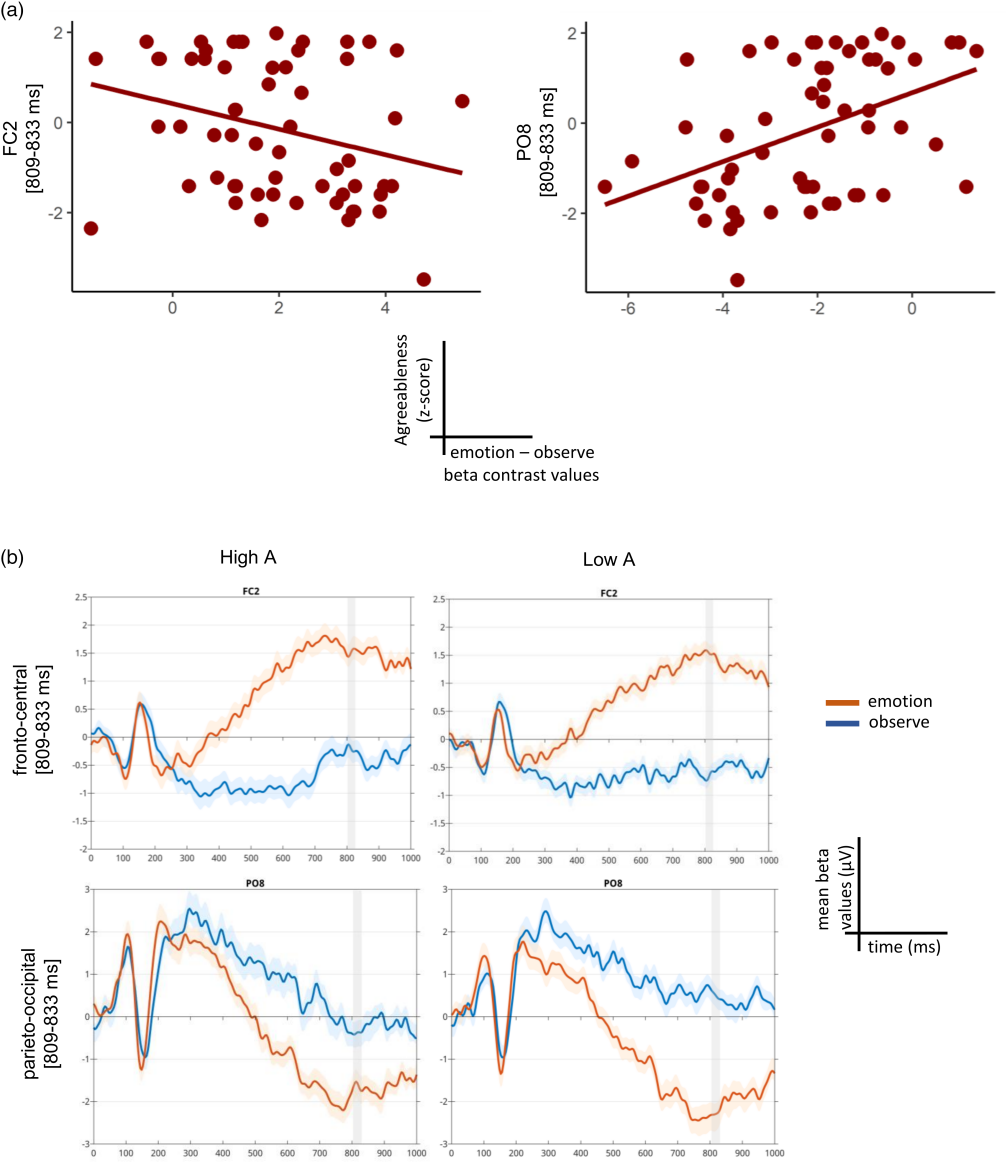
Event‐related potential (ERP)‐agreeableness skipped Pearson correlation results for the entire sample. (a) Scatterplots representing the correlation between the beta contrast values averaged across the significant time window (809–833 ms) and the agreeableness trait, separately for the most significant electrodes (FC2: max *r* = −.46, uncor *p* = .02 at 813 ms; PO8: max *r* = .54, uncor *p* = .001 at 817 ms). (b) Trace plots showing the mean beta values for the emotion (in red) and observe conditions (in blue) from each of the two electrodes, separately for the high A and low A group.

The skipped Pearson correlation between agreeableness and the ERP emotion—observe contrast did not reveal significant results when correcting for multiple comparisons within the entire sample, and either in females or males. However, we report the uncorrected results of all subjects since they align with our hypothesis that agreeableness might affect the processing of emotional stimuli also when subjects are asked to simply observe without taking action. Indeed, subjects with higher agreeableness scores had contrast values approaching to zero while those with lower agreeableness showed more positive contrast values over the fronto‐central electrodes and, as a consequence of the dipole effect, more negative contrast values over the parieto‐occipital electrodes, mostly in a time window between 809 and 833 ms (Figure 8a). These results were led by a reduced difference between the emotion and observe condition within the high A group with respect to the low A one in which the distinction between the two conditions was higher (both in fronto‐central and parieto‐occipital electrodes. Figure 8b).

#### 3.2.2 Biological (e)motion

**FIGURE 9:**
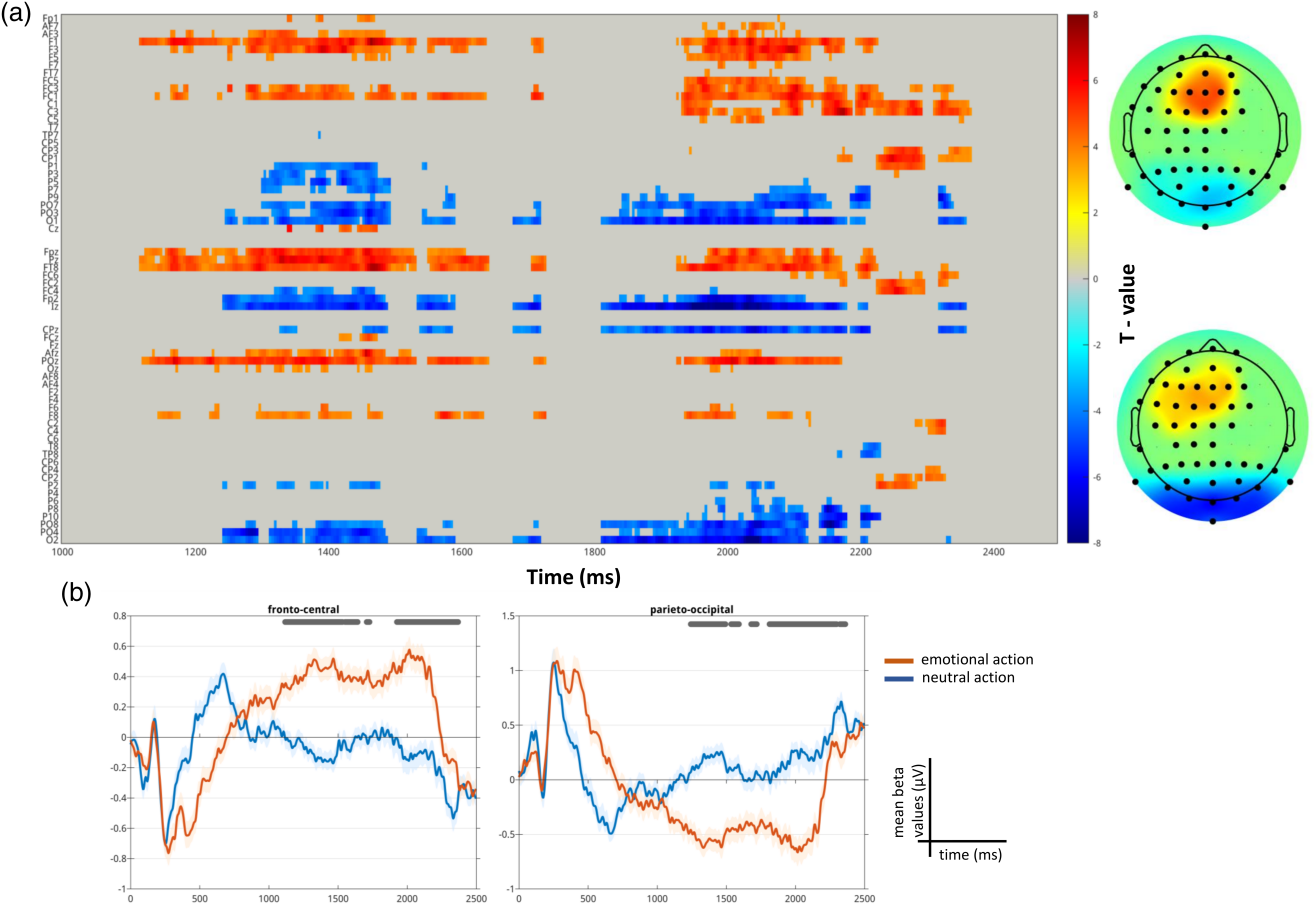
Event‐related potential (ERP) results from the paired *t* test (emotional action—neutral action), biological (e)motion (BM) task. (a) Raster plot depicting significant channels and time‐points for the paired t‐test colored based on their *t* values. On the right, the topographical representations of the *t* values averaged across the significant time‐window for the two temporal clusters (the first on the top, the second on the bottom). (b) Trace plots showing the mean beta values for the emotional action (in red) and neutral action conditions (in blue) averaged over the significant electrodes.

Two significant temporal clusters emerged from the paired *t* test between the ERP responses associated with emotional action and neutral action (cluster 1 starts at 1117 ms and ends at 1731 ms, max *t* value 7.45 at 1465 ms on channel F1, corrected *p* value <.0001; cluster 2 starts at 1813 ms and ends at 2371 ms, max *t* value 7.2 at 2175 ms on channel C3, corrected *p* value <.0001; Figure 9a). This difference was driven by the emotional condition eliciting a higher neural response (larger late positivity) than the neutral condition in the abovementioned time window over fronto‐central electrodes (Figure 9b).

However, when we performed the skipped Pearson correlation between agreeableness scores and the ERP emotional action—emotional‐neutrally action contrasts, no results survived after controlling for multiple comparisons within the entire sample, and either in female or male samples.

### 3.3 ERSP results

**FIGURE 10:**
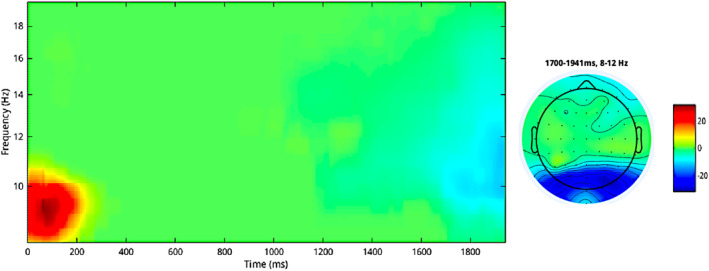
Event‐related spectral perturbations (ERSP) results from the paired *t* test (emotional action—neutral action), biological (e)motion (BM) task. Time–frequency plot of average frequencies from 8 to 20 Hz across all the 64 channels. On the right, the topographical representation of the *t* values averaged across the significant time‐window, frequencies and electrodes.

When performing the paired *t* test between the ERSP responses associated with emotional action and emotional‐neutrally action conditions, we replicated the results of Siqi‐Liu et al. (2018) showing a μ‐alpha suppression in the canonical 8–12 Hz over the centro‐parietal‐occipital electrodes (CP5, P3, P7, PO7, PO3, POz, PO4, O2) in a late time window between 1700 and 2000 ms (max *t* value —6.88, at 19141 ms, on channel PO4, corrected *p* value .0061; Figure 10).

However, same as for the ERP data, when the skipped Pearson correlation was performed between agreeableness and the ERSP contrast values in the electrodes and the averaged range of frequencies emerged from the paired *t* test (8–12 Hz), no results survived after controlling for multiple comparisons within the entire sample, and either in female or male samples.

### 3.4 Source localization

Sources were estimated only for the entire and male sample of the RME task since in females, the modulation of agreeableness was negligible and no modulation was found either for the ERP or for the ERSP data of the BM task.

**FIGURE 11:**
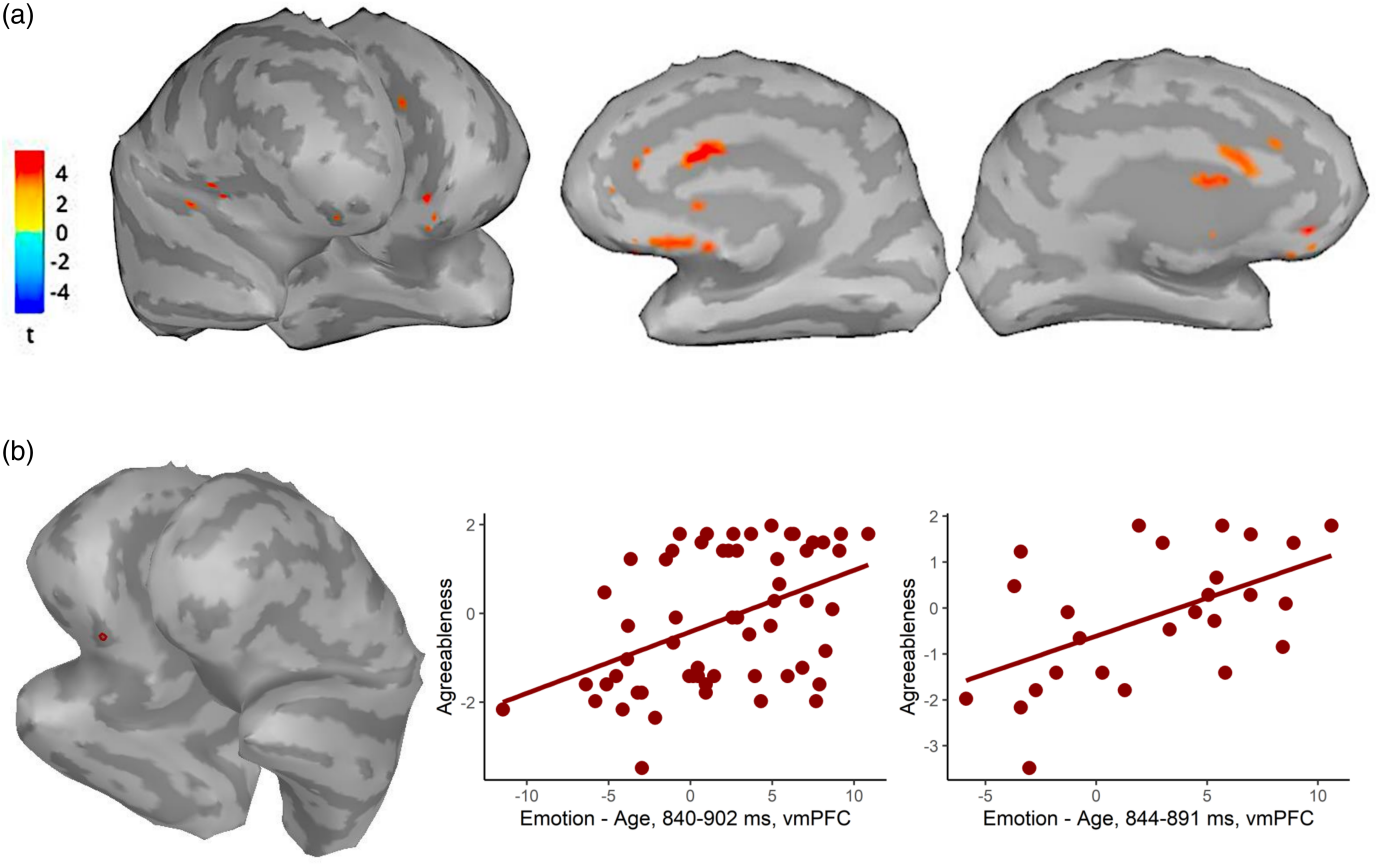
(a) Results from the paired *t* test (emotion—age) performed on sources within the entire sample, reading the mind in the eyes (RME) task. (b) Results from the skipped Pearson correlations between agreeableness and source activations. On the right the ventromedial prefrontal cortex (vmPFC) source. On the left, scatterplots representing the correlation between agreeableness z‐scores and the emotion—age contrast values in the vmPFC for the entire and male samples (respectively, left to right).

Since, within the entire sample, three different clusters emerged from the ERP effects associated with agreeableness, we analyzed separately the sources underlying these clusters and their correlation with agreeableness. To this purpose we carried out three paired *t* tests (one for each ERP cluster), and used the significant resulting brain areas as ROIs to perform the skipped Pearson correlation with agreeableness scores. Among all the ROIs that emerged from the first two paired *t* tests (ERP clusters 1 and 2), none survived the correction for multiple comparisons after the skipped Pearson correlation with agreeableness. Conversely, a significant positive correlation emerged between agreeableness and the activation for the emotion—age contrast in the ventromedial prefrontal cortex (vmPFC; *r* = .444, corrected *p* = .018; Figure 11a,b) in the averaged time window of 840–902 ms (ERP cluster 3). Thus, the source activation in the vmPFC was greater for emotion versus age stimuli in more agreeable subjects.

Noteworthy, we found a significant positive correlation between agreeableness and the activation for the sources contrast values (emotion—age) in the same ROI (vmPFC; *r* = .53, corrected *p* = .036; Figure 11b) within the male sample in a similar time window (848–887 ms), corresponding to the one of the ERP effects that were found to be associated with agreeableness. In line with the whole sample results, the source activation in the vmPFC was greater for emotion versus age stimuli also in more agreeable males.

## 4 DISCUSSION

Agreeableness is a personality trait that has consistently been associated with social cognitive processes including ToM (DeYoung & Allen, 2017; Nettle & Liddle, 2008), as evidenced by recent neuroimaging studies revealing shared neural networks (Allen et al., 2017; Udochi et al., 2022). Individual variations in agreeableness were observed to be linked to differences in social information processing within the dmPFC, a key region of ToM. More agreeable individuals encoded social and non‐social information in a more dissimilar fashion than less agreeable individuals, whose encoding of the two contents was more alike (Arbula et al., 2021).

To better understand what social information components are related to agreeableness, in a previous EEG study, we focused on the perception of social interactions, examining whether and at which stage agreeableness affects the underlying neural processes. We demonstrated that agreeableness was associated with differences in processing of social and non‐social stimuli, occurring relatively early in time. This modulation was more consistent in male participants, and was also predictive of their performance (Pisanu et al., n.d.).

Since social stimuli are often intertwined with emotional aspects, in the present EEG study, we set out to investigate the role of agreeableness in modulating emotional information processing associated with ToM and its potential interaction with the complexity of the task and the underlying neural processes. To this purpose, we selected the RME task and the BM task, both targeting emotional cues decoding, albeit with different degrees of complexity. As the former requires the decoding of high mental states from eye expressions, and the latter emotional perception processes for discriminating basic emotions from point‐light bodily stimuli, agreeableness was expected to modulate the neural responses associated with emotional versus non‐emotional conditions more strongly in the RME task and also later than in the BM task. Further predictions involved the role of sex in differentially modulating the relationship between agreeableness and emotional information processing (Pisanu et al., n.d.), and heightened sensitivity to gaze stimuli in individuals higher in agreeableness, even during passive observation.

The main results of the present study are the following. First, agreeableness modulated higher processes of mental state decoding, as tapped by the RME task, but not the emotional perception processes as assessed by the BM. In particular, individuals with higher agreeableness scores showed a tendency toward more accurate mental state recognition, compared to the control condition, although this trend did not reach statistical significance. In terms of neural responses, we found that agreeableness modulated ERP signals in a later time window, after the end of the stimulus presentation (~700–900 ms). However, when we analyzed male and female samples separately, no significant effects that lasted > = 20 ms were observed in females, suggesting that the modulation of agreeableness on the neural response in our female sample was negligible. In contrast, a later and larger cluster (844–891 ms) emerged in the male sample.

Both as a whole, and in the male sample, there was a consistent pattern in the effect of agreeableness on neural electrical activity. Participants with higher agreeableness scores exhibited a significantly higher difference between the ERPs associated with the two conditions, particularly driven by a heightened neural responsivity to emotional trials compared to non‐emotional trials, which was reduced in less agreeable participants. This is consistent with the literature reporting that a late slow ERP component over frontal regions is associated with ToM processing (e.g., Liu et al., 2004; Meinhardt et al., 2011; Sabbagh & Taylor, 2000; Tesar et al., 2020; Wang et al., 2008; Zhang et al., 2009).

Second, source estimation procedures identified the vmPFC as a brain region candidate underlying the association between agreeableness and the emotional—non‐emotional ERP effects. Specifically, highly agreeable individuals exhibited a stronger activation of this brain region when decoding mental states than in the control task (age decoding). This result was confirmed for the significant clusters observed both in the entire sample and in males.

Third, and in line with our hypotheses, we observed an association between agreeableness scores and differences in processing gaze stimuli during passive and active observation, such that individuals with high agreeableness exhibited similar neural responses during passive observation of gaze stimuli and decoding of mental states. In contrast, a greater differential neural response between the two conditions emerged in less agreeable participants. This result should be interpreted with caution, as it did not survive correction for multiple comparisons, however it might suggest that individuals with higher levels of agreeableness may decode mental states spontaneously, even during passive exposure to gaze stimuli. This interpretation is in line with research indicating that individuals with higher levels of agreeableness tend to show a heightened orientation toward social stimuli, particularly those that carry affective and affiliative connotations (Moore et al., 2014; Pisanu et al., n.d.; Wilkowski et al., 2006). Future studies are needed to confirm this result.

Taken together, these findings suggest that personality modulates the decoding of emotional cues and that this modulation seems to depend on task complexity. Indeed, the RME task constitutes the first stage of ToM processing (Baron‐Cohen et al., 2001), requiring the recognition of considerably more complex mental states, solely through static stimuli capturing individuals' gaze; in contrast, BM task requires discriminating basic emotions, primarily engaging neural perceptual processes from point‐light bodily movements that allow a quick perception and categorization of social information and emotions (Atkinson et al., 2004; Dittrich et al., 1996). In light of this evidence, we could assume that lower‐order processes tapped by the BM might not be involved in personality, since they are more automatic and require fewer cognitive resources. On the other hand, agreeableness seems to foster a marked distinction in neural responses associated with the decoding of mental versus non‐mental states. This evidence is consistent with a higher responsivity and a more in‐depth processing of emotional stimuli for effective mental states decoding in more agreeable subjects, as indicated by the increased late slow wave (LSW) observed during emotional trials, and even during passive observation of gaze stimuli. This wave has traditionally been recognized as one of the ERP components associated with cognitive aspects of ToM (see Sabbagh, 2013 for a review); however, our study provides evidence of its involvement even in affective aspects of ToM. Furthermore, the LSW has been associated with emotional encoding by facilitating the integration of emotional and cognitive information, emotional intensity (Hajcak et al., 2009; Olofsson et al., 2008; Schupp et al., 2000; Tesar et al., 2020; Yick et al., 2015; see Hajcak et al., 2010 for a review), and also used as an index of abnormal emotional responding (Dennis & Hajcak, 2009; Horan et al., 2010; Marissen et al., 2010).

Our interpretation is consistent with the involvement of the vmPFC observed from source estimation procedures, although it should be taken with caution since individual anatomies were not available. This brain region, which is highly connected to the amygdala, together with the TPJ, represents the core region of self‐other distinction and affective ToM, and in particular, it has been associated with the decoding of others' emotional states, thus with the RME task (Sabbagh et al., 2004; Schurz et al., 2014, 2021; see the entire volume by Baron‐Cohen et al., 2013).

As a fourth and last point, the sex of participants seems to modulate agreeableness, similarly to what we observed in our previous EEG study on the decoding of social interactions wherein the modulation of agreeableness lasted longer in males than in females (Pisanu et al., n.d.). This finding supports our hypothesis that personality may play a role in processing social stimuli differently in males and in females. More specifically, our results align with the sex differences observed in the social domain, where females tend to be more socially oriented and are often described as possessing inherent sensitivity to nonverbal social and emotional cues, (Baron‐Cohen et al., 2005; Hall & Halberstadt, 1981; Paletta et al., 2022; Pavlova et al., 2010; Proverbio, 2021; Proverbio et al., 2008; Stake & Eisele, 2010). This inherent sensitivity likely contributes to the lacking impact of personality on emotional cue decoding observed in females. This examination contributes to refining social cognition models, offering insights into the much‐discussed distinctions between males and females, preventing gender bias in research to obtain nuanced results applicable to diverse populations, and facilitating the adoption of effective interventions, particularly in clinical psychology. In addition, considering sex differences contributes to understanding the evolutionary roots of personality traits and their potential adaptive functions in males and females.

However, when the behavior was considered, only a weak modulation of agreeableness emerged in the sample as a whole. This may be partially because we recruited healthy participants from the general population, without any overt deficit affecting their emotional processing abilities. Agreeableness may still influence the neural process underlying mental states recognition, even without affecting the ability to recognize them, as observed also in our previous EEG study, wherein the modulation of agreeableness at the behavioral level was weak (Pisanu et al., n.d.). Although a similar null result between agreeableness and RME performance was reported by Nettle and Liddle (2008), which led them to conclude that this personality trait is unrelated to the affective component of ToM associated with mental state decoding, findings from our current study challenge this assertion, suggesting a different perspective.

The study of agreeableness has a profound impact on various aspects of life. Directly associated with positive outcomes in areas such as interpersonal relationships, individual fulfillment, life satisfaction, and mental health, agreeableness emerges as a determinant of overall well‐being (Allen & DeYoung, 2017; DeYoung & Blain, 2020; Tackett et al., 2019). Delving deeper into the cognitive mechanisms of agreeableness, particularly its association with ToM, can improve our predictive abilities and provide deeper insights into different behaviors and interpersonal relationships. Exploring how the brain works in agreeable individuals who show kindness, empathy, altruism, opens avenues for improving social cognitive skills ranging from educational strategies to pharmacological manipulation and tailored clinical interventions that consider interindividual differences in the design of goals and methodologies. Furthermore, it becomes essential to reveal how normal functioning differs in psychopathologies characterized by social deficits and to outline the impact of individual differences in social cognition on the success of relationships in the real world, on the quality of the social network, and on interpersonal functioning. Overall, the study of agreeableness proves crucial for deciphering the complexities of human interaction and paving the way for interventions that promote positive social outcomes.

Although our study provides valuable insights into the domains of personality and social cognition, it is imperative to recognize some limitations that shape the interpretation and generalizability of our findings. First, our findings lack direct comparisons in existing studies, needing validation through future research. Second, the null results in the BM task do not definitively indicate the absence of agreeableness modulation in the neural response. Therefore, further tests are warranted to ascertain whether its modulation specifically occurs in tasks involving high‐level neural processes. Furthermore, our exploration of the modulation of agreeableness on μ‐suppression is incomplete, limited exclusively to the BM task where we found no significant results. In this regard, we cannot definitively state that the observed effects of the task itself exclusively represent μ‐suppression since there is a possibility that an alpha effect, particularly involving the occipital electrodes, may also be at play.

In summary, to fully investigate the association between agreeableness and μ‐suppression, other alternative tasks and/or stimuli within the Eyes task should be included, such as those used in Pineda and Hecht's (2009) study. Finally, the inconsistency between behavioral and neural findings could be considered another limitation of the current study. To this regard, we emphasize that behavior is influenced by many factors and, while EEG is sensitive in capturing subtle brain differences in healthy individuals, accuracy and RT measures are not. Furthermore, designing tasks with a dichotomous yes/no response format may limit capturing variability and contribute to the observed inconsistency. Interestingly, our tasks were not designed to weigh on RTs, and this is likely another reason why we failed to observe behavioral differences with this measure. Exploring swifter tasks that require instantaneous responses could provide an interesting avenue to uncover distinctions, perhaps not in terms of accuracy, but rather regarding delayed responses between individuals.

## 5 CONCLUSIONS

The current study expands our understanding of the role of personality in social skills by revealing how neural mechanisms underlying emotional cues decoding are modulated by the agreeableness trait as a function of task difficulty and sex. Our results show that agreeableness affects brain's activity during mental state decoding in male participants, while in females this modulation seems to be negligible. Considering the limited body of research on this particular topic, further replication studies are needed to better understand the role of personality in emotional processing in both healthy and clinical populations and its effects on behavior. Overall, these findings offer significant insights into the field of individual differences within the social domain, holding potentially useful implications for clinical interventions aimed at addressing social impairments, developing customized interventions that account for individual variations, thus contributing to the advancement of tailored and effective therapeutic approaches.

## FUNDING INFORMATION

This research did not receive any specific grant from funding agencies in the public, commercial, or not‐for‐profit sectors.

## CONFLICT OF INTEREST STATEMENT

The authors have no known conflict of interest to disclose.

## Supporting information


**DATA S1.** Supporting Information.

## Data Availability

The data that support the findings of this study are openly available in OSF at https://osf.io/xauev/?view_only=d31e44d130134f38baba840ff3c49b95.
