# Supplementary material for: Agreeableness modulates mental state decoding: Electrophysiological evidence
Source: Hum Brain Mapp. 2024 Jan 30;45(2):e26593. doi: 10.1002/hbm.26593 (PMC10826893; doi:10.1002/hbm.26593)
Supplement: Supplementary file 1 — DATA S1. Supporting Information. [file HBM-45-e26593-s001.docx]

Supplementary material

Behavioral Pilot – RME task

The behavioral pilot involved 31 participants (18-35 years, 23 females). After controlling for the normality of the data, two paired t-tests were performed between the emotion and age conditions separately for accuracy and RTs in R v.4.1.1 (R Core Team, 2020). The two conditions differed significantly in terms of accuracy (*t* = 6.085, df = 30, *p* < .001) with participants being less accurate in the age condition compared to the emotional condition. No significant differences were found for RTs (*t* = -0.57, df = 30, *p* = 0.57).

Therefore, the age condition presented a greater level of difficulty compared to the emotion condition. This deliberate design aimed to rule out potential confounding effects arising from task complexity for the EEG study. Indeed, in contrast to other studies that commonly adopt the sex of depicted subjects as a control condition, which tends to be comparatively straightforward, our intentional choice of a more challenging condition enhances the robustness of our findings.
